# Mortality and morbidity patterns in under-five children with severe acute malnutrition (SAM) in Zambia: a five-year retrospective review of hospital-based records (2009–2013)

**DOI:** 10.1186/s13690-015-0072-1

**Published:** 2015-05-01

**Authors:** Tendai Munthali, Choolwe Jacobs, Lungowe Sitali, Rosalia Dambe, Charles Michelo

**Affiliations:** Department of Public Health, School of Medicine, University of Zambia, PO Box 50110, Lusaka, Zambia; Department of Biomedical Sciences, School of Medicine, University of Zambia, Lusaka, Zambia; Ministry of Ministry of Community Development Mother and Child health, Private Bag W252 Sadzu Road, Lusaka, Zambia

**Keywords:** Severe acute malnutrition, HIV, Mortality, Under-five children, Zambia, Hospital, Comorbidity

## Abstract

**Background:**

Severe acute malnutrition has continued to be growing problem in Sub Saharan Africa. We investigated the factors associated with morbidity and mortality of under-five children admitted and managed in hospital for severe acute malnutrition.

**Methods:**

It was a retrospective quantitative review of hospital based records using patient files, ward death and discharge registers. It was conducted focussing on demographic, clinical and mortality data which was extracted on all children aged 0–60 months admitted to the University Teaching Hospital in Zambia from 2009 to 2013. Cox proportional Hazards regression was used to identify predictors of mortality and Kaplan Meier curves where used to predict the length of stay on the ward.

**Results:**

Overall (n = 9540) under-five children with severe acute malnutrition were admitted during the period under review, comprising 5148 (54%) males and 4386 (46%) females. Kwashiorkor was the most common type of severe acute malnutrition (62%) while diarrhoea and pneumonia were the most common co-morbidities. Overall mortality was at 46% with children with marasmus having the lowest survival rates on Kaplan Meier graphs. HIV infected children were 80% more likely to die compared to HIV uninfected children (HR = 1.8; 95%CI: 1.6-1.2). However, over time (2009–2013), admissions and mortality rates declined significantly (mortality 51% vs. 35%, P < 0.0001).

**Conclusions:**

We find evidence of declining mortality among the core morbid nutritional conditions, namely kwashiorkor, marasmus and marasmic-kwashiorkor among under-five children admitted at this hospital. The reasons for this are unclear or could be beyond the scope of this study. This decline in numbers could be either be associated with declining admissions or due to the interventions that have been implemented at community level to combat malnutrition such as provision of “Ready to Use therapeutic food” and prevention of mother to child transmission of HIV at health centre level. Strategies that enhance and expand growth monitoring interventions at community level to detect malnutrition early to reduce incidence of severe cases and mortality need to be strengthened.

## Background

Globally about 25 to 35 million under-five children have severe acute malnutrition (SAM) and 13 million of these children live in sub-Saharan Africa and of these children one million will die every year [1]Severe acute malnutrition, is characterised by wasting (marasmus), oedema (as a result of kwashiorkor), or both (marasmic kwashiorkor), and occurs mostly in children [2]. Marasmus is diagnosed when subcutaneous fat and muscle are lost because of the body’s process of mobilising energy and nutrients. Clinical features usually include a triangular face, extended abdomen (from muscular hypotonia) and anal or rectal prolapse (from loss of perianal fat). Features such as oedema, changes to hair and skin colour, anaemia, hepatomegaly, lethargy, severe immune deficiency and early death characterise kwashiorkor [2]. These and many other complications partly explain why malnutrition continues to be a major cause of disease burden especially in low-income countries killing millions of children [3-8].There is evidence illustrating how substantial investments have been implemented to avert associated negative effects [9,10]. However many developing countries continue to experience poor child growth rates, high morbidity and mortality with about three million child deaths due under nutrition every year [11].

A vast majority of children suffering from acute malnutrition are found in Sub Saharan Africa (13 million, 9.4% of all under fives in the region). The worst affected in Africa are west, central and eastern Africa [1]. In Zambia, about 5% of children have severe malnutrition, 16% are underweight and 45% are stunted and it has been suggested that this accounts for up to 52% all under-five deaths [12,13] Under-five severe malnutrition rates have been high for the past decade [14]. In view of this, the Ministry of Health in Zambia has since 2005, implemented the Integrated Management of Acute Malnutrition programme which looks at community and health centre-level management of uncomplicated severe acute malnutrition and training of health workers [13]. The programme is a community-based approach for the management of uncomplicated severe acute malnutrition where Ready-To-Use Therapeutic Foods (RUTF) are made available to families of children with severe acute malnutrition through either a health facility or a community health worker [8,13,15]. Community health workers are trained to identify children with SAM and to recognise those children who need urgent treatment or have complications and need referral [15]. At hospital level, training of health care workers in World Health Organization (WHO) treatment guidelines is part of the policy to manage complicated SAM in the in-patient setting. The WHO treatment guidelines are a ten-step protocol with three phases of treatment, which include stabilisation, rehabilitation, and discharge and follow up used in inpatient settings in the country [16].

However, a nation-wide increase in the levels of admission for children with SAM was noticed in the last quarter of the year 2008 in a study by the Nation Food and Nutrition Commission NFNC in 2008 [17]. This was despite the promotion of community therapeutic care for treatment of uncomplicated severe malnutrition and improvements in early identification of malnutrition and reduction of congestion in hospitals [8,17]. The University Teaching Hospital (UTH) recorded up to 50% mortality rates in the last quarter of 2008 [17]. Similarly, Trehan I et al. [18] also revealed that at UTH, case fatality rates were about 40%. Irena et al. [19] conducted a cohort study involving children 6–59 months old with SAM admitted to the UTH as in-patients. The study revealed that overall mortality rate was at 40% while at 48 hours after admission it was 30.6% and 65% at 1 week of admission. This information clearly suggests there might exist unexplored dynamics that could explain in part or in full the reasons for this high burden. Although this could be multi-factorial, one expects that at hospital level, there exists an environment that should be examined easily when such questions arise. We hypothesised that mortality and morbidity rates are still high and in this regards present study aimed to determine morbidity and mortality patterns and to investigate factors that could be associated with severe acute malnutrition among under five children attending UTH from 2009 to 2013.

## Methods

### Population and sampling procedures

This study was done at UTH in Lusaka, Zambia in the Nutrition Rehabilitation Unit. The Nutrition Rehabilitation Unit only admits children under 14 years and has admissions of complicated SAM from all around the country. The unit has a bed capacity of 59 and admits all year round. Owing to high numbers of cases of complicated SAM than the unit can accommodated cot sharing is a common practice. The ward is managed by three rotating resident physicians, two junior resident medical officers, a senior registrar, a consultant paediatrician and three to five nurses. All admissions are examined by the attending physician and comobidities were assessed clinically by attending physicians. All the children are managed using WHO standard guidelines for the management of SAM.

In the first phase F75 therapeutic milk (prepared using fresh or fermented milk on the ward) are used to nutritionally rehabilitate the children. After the nutritional rehabilitation F100 therapeutic milk or a peanut based ready-to-use therapeutic food (RUTF) is used to stabilize the children. Once the child is stabilized, they are discharged and referred to one of the outpatient therapeutic programs (OTP) for full recovery. HIV serology for children older than 19 months of age was done using Determine® HIV-1/2 test while for children 18 months and younger DNA PCR was done after obtain consent from caregivers. All children found to be HIV infected are commenced on ART in the wards and on discharge are referred to one of the outpatient therapeutic programs for continued management.

All 0–60 months of age children admitted to the inpatient nutrition unit (AO7) between 2009 and 2013 were eligible for the study. Admission to the unit is based on the presence of bilateral pitting oedema and /or weight for height Z-scores (WHZ) < −3 standard deviations (SD). The WHO weight-for-length reference card (growth standards charts) are used to calculate weight for height Z- scores.

The study was a retrospective quantitative review of hospital based records. The sample collected comprised some records with incomplete information especially from 2009 to 2011. Overall the *de facto* eligible sample amounted to 9540 records. Some registers and files were missing from the ward, which led to missing data for most of the variables like types of malnutrition, mortality, co-morbidity and HIV. Given that a complete case analysis method was the default setting for the software used to analyse the data in this study, a sensitivity analysis was done to ascertain whether the findings of this study would have been different if the missing data had been included in the analysis. A dichotomous variable was then created using the total number of missing and present data in the dependant variable mortality. The variable was then cross tabulated in chi square to compare whether there were more missing values than known values in the independent variables. The analysis shows that generally the known data was more than the missing. Consequently it can be inferred that the results could not have changed significantly if all the missing data were added to the analysis.

### Data extraction

Trained assistants extracted data on variables of interest from ward A07 patient death and discharge registers and files, using an Excel-based predesigned data collection form. Additional linked data on HIV infection status was also sourced from the Paediatric HIV Centre of Excellence (POCE) within UTH. Completeness of the information on all of the socio-demographic variables, clinical data including ultimate outcome (death or discharge) as recorded in the registers and legibility of each filled in data collection form were audited at the end of each day to ensure accuracy. The extracted data was then entered into conventional Microsoft Excel operating system. Data verification, coding, cleaning and validation were conducted until the database corresponded with the data collected on the collection forms after which it was exported to Stata (version 11) using Stat-Transfer software in readiness for analysis.

### Data analysis

Intercooled Stata version 11 (College Station, Texas, USA) was used for all the analyses. Descriptive statistics were used to summaries age and length of stay on the ward. To examine if morbidity, co-morbidity, residence, and HIV status were associated with mortality, Cox proportional hazards regression was performed using all the categorical exposure variables after conducting a chi-square test of independence initially. Model diagnostics were done using the maximum likelihood estimation (MLE) and the Hosmer-Lemeshow goodness-of-fit. Kruskal Wallis was used to compare if means of “length of stay on the ward” and “age” for the different types of SAM were different. Cox proportional hazards regression was used to predict the risk of death in the co-morbidity and morbidity groups while adjusting for other variables. All variables were added to the model except the continuous variables age and length of stay. Cuzick, a non parametric test for trends, was used to examine trend patterns for both morbidity and morbidity between 2009 and 2013. Kaplan-Meier curves were also used to estimate survival probability of different types of SAM. A p-value of less than 0.05 was considered as significant and 95% confidence intervals were reported. Since analyses were not done in yearly strata (2009–2013), the term “overall” was reserved for pooled estimates that covered all records of all years together.

There were various co-morbidities that were recorded in the data. These co-morbidities included respiratory tract conditions such TB, Pneumonia, bronchitis, cough, flu; skin conditions such as burns, dermatitis, abscesses, chicken pox and measles; birth defects like cerebral palsy, cleft lip pallet; blood conditions such as anaemia, septicaemia, sickle cell disease, malaria, cardiac conditions, meningitis and neoplasm among other conditions. The co-morbidities were then analysed based on frequencies and then the co-morbidities with the lowest frequencies (n < 100) were grouped together and labelled “Other”. Five comorbidity groups’ namely Anaemia, Septicaemia, Tuberculosis, Diarrhoea and Other were finally analysed as a single variable labelled co-morbidity. HIV was analysed as a separate comorbidity.

### Ethical approval

Ethical clearance (IRB No. 00005948) was granted by Excellence in Research Ethics and Science (ERES). Permission to conduct the study was solicited from UTH authorities and Ministry of Health.

## Results

### Population and demographic characteristics

Overall (n = 9540 under-five children), there were 5,148 (53.9%) males with a median age was 17 (IQR 12–22) months. Majority (93.3%) of the records were for children from the Lusaka area (the capital city) with only seven percent from out of Lusaka. The median length of stay for children who were discharged was 12 days (IQR 8 to 16 days) while for those who died was 3 day (IQR 1 to 7 days), (Table 1).

**Table 1:** **Background characteristics of under-five children with SAM attending University Teaching Hospital in Lusaka, Zambia**

**Characteristic**	**Population**	
	**n**	**%**
**Sex (n = 9 534)**		
Male	5 148	53.9
Female	4 386	45.9
**HIV status (n = 8 589)**		
Negative	5 827	67.8
Positive	2 589	32.2
**Morbidity (n = 9 076)**		
Marasmic-Kwashiorkor	1 511	16.4
Marasmus	1 957	21.6
Kwashiorkor	5 609	62.0
**Co-morbidity (n = 2 037)**		
Anaemia	218	11.6
Diarrhoea	723	29.8
Pneumonia	544	25.3
Septicaemia	146	5.3
Tuberculosis	113	6.8
Other	293	21.2
No co-morbidity	7 503	100
**Residence (n = 5 141)**		
Lusaka	4 791	93.0
Out of Lusaka	358	7.0

NOTES: 1. Sample size; Overall n = 9540 sample size was dictated by responses for age. 2. Yearly samples were 2151(2009), 2301(2010), 1999(2011), 1726(2012) and 1363(2013). 3. Median age in months was 17 months IQR (11–22); median length of stay in days was 8 days IQR (3–14); overall mortality 46.7% (2,804).

### Morbidity patterns

Kwashiorkor was seen as the most frequently recorded type of SAM accounting for 62.0% of the children. This was followed by marasmus that accounted for 21.6% of the children while Marasmic–kwashiorkor had 16.4%. Of the co-morbidity recorded (n = 2,037), 11.6% had anaemia, 29.8% had diarrhoea, 25.3 % had pneumonia, 5.3% tuberculosis and 6.8% septicaemia, while 21.2% had other co-morbidities (Table 1).

Median age among children with kwashiorkor was 18 months (IQR 12–23), 17 months (IQR12-22) for those with marasmic-kwashiorkor and 15 months (IQR 11–20) for those with marasmus only (P = 0.001). Results also showed that HIV infection prevalence was 32.2% and the bulk of this was among children with marasmus (40%) while children with kwashiorkor and marasmic kwashiorkor accounted for 30.4% and 30.5% respectively (p < 0.001). Diarrhoea was significantly more common in children with marasmic-kwashiorkor group and lowest in children with kwashiorkor (p = 0.009. While on the other hand Septicaemia had the lowest prevalence across the co-morbidity groups accounting for 5.2% in the marasmus group, 5.4% in the kwashiorkor group and 5.1% in the marasmic-kwashiorkor group (p = 0.009). Prevalence of anaemia among marasmic children was only 7.4%, significantly lower when compared to those with kwashiorkor and marasmic kwashiorkor (13.2 and 13.4 respectively (P = 0.009), Table 2.

**Table 2:** **Morbidity by demographic factors of the study population of under-five children with severe acute malnutrition attending University Teaching Hospital, Lusaka Zamb**ia

**Characteristic**	**Morbidity**			**P value** ^*^
	**Marasmus**	**Kwashiorkor**	**Marasmic-Kwashiorkor**	
	**%(n)**	**% (n)**	**% (n)**	
**Sex**				0.337
Male	52 (1 023)	54 (3 037)	54 (824)	
Female	48 (929)	46 (2 572)	46 (685)	
**Median age**	15 months	18 months	17 months	0.0001^**^
	IQR (11–20)	IQR (12–23)	IQR (12–22)	
**HIV status**				0.000
Negative	59.9 (1 050)	69.6 (3 521)	69.5 (942)	
Positive	40.1 (703)	30.4 (1 537)	414 (30.5)	
**Co-morbidity**				0.009
Anaemia	7.4 (33)	13.2 (121)	13.4 (50)	
Diarrhoea	31.4 (140)	27.6 (253)	33.6 (125)	
Pneumonia	25.8 (115)	210 (22.9)	23.4 (87)	
Septicaemia	5.2 (23)	5.4 (50)	5.1 (19)	
Tuberculosis	35 (7.8)	56 (6.1)	7.5 (28)	
Other	99 (22.2)	225 (24.6)	16.7 (62)	
**Residence**				0.200
Lusaka	91.9 (1 003)	93.5 (2 656)	93.0 (866)	
Out of Lusaka	8.1 (88)	6.5 (183)	7.0 (65)	

*Tested using Chi square.

**Tested using Kruskal –Wallis.

### Mortality, survival, determinants & patterns

**Figure 1:**
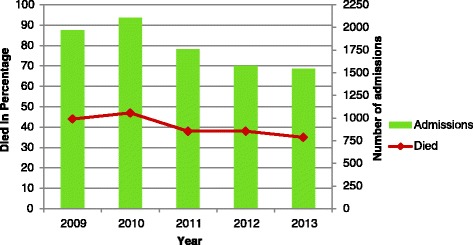
**Shows mortality trends by year and admission.**

**Figure 2:**
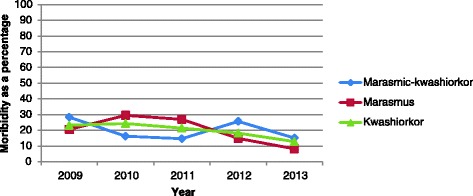
**Shows morbidity trends by year and admissions.**

Generally, the Cuzick non-parametric test for mortality trends showed a significantly decreasing pattern (P < 0.0001) and this decrease was associated with a decrease in admissions. Overall, proportion of children that died over this period was 46% and this significantly declined overtime from 51% in 2009 to 34.8% in 2013 (P < 0.05), Figures 1 and 2. This mortality pattern differed by age in that it was higher in males than females (52% vs. 48%, P = 0.007). Mortality at 24 hours, 48 hours and 1 week of admission was 93.2%, 88.7 and 29% respectively.

Admissions and mortality and morbidity rates seem to be showing a steady decline over the years being investigated. Nevertheless mortality was high in children who were HIV infected with 80% more likely to die than those who were HIV uninfected (HR 1.8, 95% CI 1.6 – 2.0, P < 0.001). While those with septicaemia were approximately 2.8 times more likely to die compared to those without comobidities (HR 2.8, 95%CI 2.9 -3.9), those with diarrhoea were 60% more at risk of dying compared to those who had no commodities (HR 1.6, 95% CI 1.3 - 1.9). Pneumonia also increased the risk of dying by 30% compared to children without any comorbidity (HR 1.3, 95% CI 1.0 – 1.6), Table 3.

**Table 3:** **Cox proportional Hazards showing risk of mortality among under-five children with severe acute malnutrition attending University Teaching Hospital, in Lusaka**

**Characteristic**	**Population**	**Mortality** ^*^	
	**%(n)**	**Hazards Ratio (CI)**	**P value** ^*^
**Sex (n = 9 534)**			
Male	53.9 (5 148)	1	
Female	45.9 (4 386)	1.1 (1.1-1.2)	0.034
**HIV status (n = 8 589)**			
Negative	67.8 (5 827)	1	
Positive	32.2 (2 589)	1.8 (1.6- 2.0)	0.000
**Morbidity (n = 9 076)**			
Marasmic-kwashiorkor	15.8 (1 511)	1	
Marasmus	21.6 (1 957)	1.1 (1.0-1.7)	0.159
Kwashiorkor	61.8 (5 609)	1.0 (1.1-1.8)	0.707
**Co morbidity (n = 2 037)**			
No comorbidity	78.7 (7 503)	1	
Anaemia	2.3 (218)	1.0 (0.8-1.4)	0.933
Diarrhoea	7.6 (723)	1.6 (1.3-1.9)	0.000
Pneumonia	5.7 (544)	1.3 (1.0-1.6)	0.008
Septicaemia	1.5 (146)	2.8 (2.9- 3.9)	0.000
Tuberculosis	1.2 (113)	1.0 (0.5-1.4)	0.971
Other	3.1 (293)	1.8 (0.7-1.8)	0.000
**Residence (n = 5 141)**			
Lusaka	50.2 (4 791)	1	
Out of Lusaka	4.0 (358)	0.7 (0.5- 0.9)	0.010

*tested using Cox proportional Hazards Regression.

**Figure 3:**
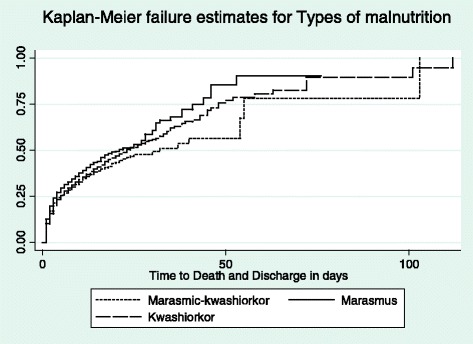
**Shows children with Marasmus were more likely to die compared to children with Kwashiorkor or Marasmic-kwashiorkor.**

Despite the differential mortality patterns largely dictated by HIV infection patterns within all the nutritional morbid states, Kaplan Meier failure curves showed that children with marasmus had increased risk of dying independent of HIV infection status, Figure 3. There were 5300 subjects considered in the survival analysis with a median survival time of 13 days. The lower quartile survival time was 3 days and upper quartile time is 19 days. Total person time at risk is 55078 days. The incidence rate was estimated at 0.056 per day or 20.4 per year. In person years this would be 20,418 per 100 person years per year. Log rank test revealed significant differences among the median time to death or discharge across the different types of SAM (p < 0.001), Table 4.

**Table 4:** **Median length of stay for both deaths and discharges among under five children with severe acute malnutrition attending University Teaching Hospital in Lusaka Zambia**

**Characteristic**	**Died Median(ICR) %(n)**	**Discharged Median(ICR) %(n)**	**Log rank P value**
Marasmic- kwashiorkor	2 (1–6) 42% (439)	12 (8–17) 58% (609)	0.0000
Marasmus	2 (1–7) 51% (659)	12 (8–17) 49% (626)	
Kwashiorkor	3 (1–7) 55% (849)	12 (8–16) 45% (1, 526)	

## Discussion

A significantly high but declining burden of severe acute malnutrition comprising the core morbid nutritional conditions, namely kwashiorkor, marasmus and marasmic-kwashiorkor still exist in differential proportions as observed in hospital records of under-five children admitted to the University Teaching Hospital between 2009 and 2013 in Zambia. This decline was associated with declining admissions which is further associated with declining mortality. HIV-related mortality was also found to have contributed substantially to the overall burden as it worsened the picture by increasing the number of comobidities. Other core co-morbidities explored in this review included tuberculosis (TB), anaemia, diarrhoea and septicaemia and how they are associated with patterns of mortality and morbidity in under-five children with kwashiorkor, marasmic kwashiorkor marasmus.

The data used in this study was collected amidst several strengths and limitations which could have biased the results. Firstly, data quality was a limitation because it is recorded from setting where there are high levels of staff turnover and fatigue, ward congestion, and limited diagnostic capabilities which led to missing information in both files and registers. In view of the missing data a sensitivity analysis was conducted which revealed that the results could not have changed significantly if all the missing data were added to the analysis. Nonetheless, the sample size in this study is sufficient and data collected covers five years which is representative of the UTH as a study setting and thus reduces the likelihood of any results derived from analysis being due to chance or situational variations. Moreover, since the data was sourced from UTH which receives referrals from hospitals in every province in the country, the findings could be a representation of morbidity and mortality in referring hospitals in the country. However mortality could be slightly higher at UTH as only very severe cases are referred there. In addition follow up of discharged children is not done at the hospital but at community based health facility level which means some children are lost to follow up because their caregivers will not report to the nearest health centre for continued care after discharge and so full recovery could not be reported in this study. Furthermore, the findings of this study are descriptive in nature, it is therefore important to consider explorative studies to be carried out to ascertain why trends and pattern of mortality, morbidity and co-morbidity are they way they are in ward AO7.

It was not surprising to find that kwashiorkor is still the most common morbidity and had the highest presence of other morbid conditions such as septicaemia and anaemia. One of the reasons could be that the staple food in Zambia (maize) is carbohydrate based and most protein rich foods may not be available in required quantities putting families at risk of this type of malnutrition given cost related financial constraints associated with proteins. According to Latham [20] the incidence of kwashiorkor has been associated with high carbohydrate intake and inadequate protein intake [21]. This has been outlined in other studies targeting this population and the region where carbohydrate based foods are also staple foods [19,22,23]. Fuchs [24] further argued that kwashiorkor in these regions is primarily due to inadequate protein diet, aflotoxcins in food, inadequate weaning and infection. The median age of occurrence of kwashiorkor that was found in this study coincides with the weaning period (18 months) generally practiced in this population [17]. This also coincides with the period when the intake of macro and micro nutrients is reduced in these children and this could partly explain why nutritional anaemia among other nutritional disorders are common as observed in this study.

Surprisingly in this study, those who had kwashiorkor also had the lowest prevalence of pneumonia and lowest prevalence of HIV infection. This confirms earlier reports by Amadi et al. [25] who reported that kwashiorkor was more associated with HIV uninfected children in this population. The reasons for this are unclear and were beyond the scope of this study but need further exploration particularly given that the opposite is what is expected as both HIV and kwashiorkor significantly reduces ones humoral immunity.

The association between HIV and marasmus was however different. Children who were diagnosed as having marasmus were also found to have the highest prevalence of HIV infection. Marasmus and HIV infection in children were recorded to have presented with severe wasting which could be due to the body’s physiologic mechanisms of adapting to lower food intake which becomes pronounced in HIV infected children due to opportunistic infections such are oral candidiasis that make food intake difficult [18,21]. It was also revealed by Amadi et al. [25] that marasmus was the predominant form of malnutrition amongst HIV infected children, in line with findings in this study. In general we have reported that children who were HIV infected were 80% more likely to die than those who were HIV uninfected. Further we also observed from Kaplan Meier survival curves and the log rank test that having marasmus or indeed marasmic-kwashiorkor reduced the survival chances of the children. Although the contribution HIV has is well documented, we also know that available evidence shows that in general HIV related mortality is declining. It may be reasonable to then argue that the bulk of the mortality burden observed in severely malnourished children might be reduced if strategies to prevent or manage the HIV part are targeted. We also know that childhood HIV is still a huge problem in Sub-Saharan Africa.

We also observe further that Heikens et al. [26] revealed that in Sub Saharan Africa mortality is three times higher in HIV infected children than in HIV uninfected children but given that this was not stratified by nutritional morbidities, we postulate further that it is possible that some of this could be due to marasmus or marasmic effects. We argue thus, notwithstanding available evidence outlining that because HIV infected children with SAM have different pathophysiology, case management and referral pathways compared to HIV uninfected children, this in itself makes therapeutic and palliative care very difficult increasing the likelihood of mortality[26]. In this regard, Heikens et al. [26] further argued that there is urgent need for setting specific therapeutic guidelines based on evidence from high HIV burden regions for such morbidities.

Other than HIV and anaemia, core comobidities explored in this review included diarrhoea, pneumonia and septicaemia. Children who were diagnosed with TB and those with septicaemia were also more at risk of dying compared to those who had anaemia, respectively. Although comorbidity stratified analysis were not reported in this study, we still argue that this is plausible because high HIV prevalence among children found with TB and septicaemia has been reported elsewhere in the region. Asafo-Agyei et al. [27] and Ashworth et al. [28] in two hospitals in South Africa also found an association between septicaemia and high mortality especially in the HIV infected children.

Despite the reported burden of comobidities associated with severe acute malnutrition, mortality and morbidity trends are declining. The reasons for this were beyond the scope of this study. However we are aware that there are numerous interventions that have been put in place that could be associated with the decline at community level to combat severe acute malnutrition and this has been coupled with similar facility based interventions such as the provision of RUTF at health centre level. Heikens et al. [26] argued in similar lines that RUTF aids in effective home-based management of uncomplicated SAM leading to recovery rates of 90 per cent and five per cent mortality rate. This then results in approximately only ten percent admissions from the communities. If this hypothesis holds, it could partly explain the declining admissions observed over the period report in this study. This could also have been partly attributed to general reported reducing prevalence of malnutrition in developing countries of about 38 to 25 percent between periods of 1980 and 2000 due to improvements in lifestyles [29]. This is supported by global reports by UNICEF, WHO, and World Bank [1] arguing that global rates of acute malnutrition have reduced by approximately 11% in the past 25 years. In this regard and although overall mortality was found to be higher in this study compared to other studies [18,19,30], the general pattern was found to be declining and 3.3 percent lower than the 50 percent reported by National Food and Nutrition Commission (NFNC) in 2008 [17].

We note that this burden is a reflection of accumulations of malnutrition itself, associated and various morbid conditions as well as mortality, notwithstanding that over time, the population demographics might change considerably too. Trends therefore reflect a time averaged dynamic balance between incidence of core and associated morbidities, migration and mortality. The observed decline in burden could thus have been influenced by any or a combination of these factors. However, we realise that the changes were generalised. HIV related mortality’s contribution to overall burden changes might reflect the impact of associated care and support strategies as well as improved access to anti-retroviral therapy for HIV which have prolonged life expectance in both adults and children.

## Conclusions

The study was conducted to provide information on factors associated with morbidity and mortality associated with under-five children admitted with SAM at UTH from 2009 to 2013. We find evidence showing a gradual decline in mortality and morbidity. This decline could be attributed partly to interventions that the country has put in place in addition to the improvements in socio-economic situation projected in the last 25 years. Finding critical associations with HIV infections suggest an integration of preventive measures that combine HIV testing with child survival strategies. It seems reasonable to argue that the test and treat strategies be widened in scope for both pregnant and breast feeding mothers, and those found positive be put on treatment to reduce mother to child transmission of HIV. In addition, the indication that community interventions could be working further suggests that primary preventive child survival strategies be intensified with particular focus on sensitization mothers with under-five children regarding proper child feeding practices. An integrated approach earlier suggested could include but not limited to spreading the social cash transfer scheme to parents of malnourished under-five children to increase intake of protein rich foods and reduce the risk of developing kwashiorkor. This needs to be done whilst ensuring continuity and sustainability of the RUFT in the health facilities to reduce incidence of severe acute cases of malnutrition. If these strategies are integrated coupled with integration of operational research strategies to continually create evidence based information for adjusting these strategies, Zambia could record immense successes in reducing overall and all cause child health morbidity and mortality, and consequently increase the quality of life for its children, a desire and vision that is attainable.

The problem of SAM is a complex study and this review presented a descriptive picture of patterns of mortality in an inpatient setting in children with different types of SAM and associated comobidities. There remains a vast array of answered questions on why SAM trends are declining, what the role of nosocomial infections is in mortality patterns, how WHO guidelines are implemented and the use of antibiotics in management of with SAM in Zambia among other question. It is hoped that future research on SAM in Zambia would address some of these questions.

### Supporting information

Data analysed for this article.

## Abbreviations

HIV: Human immunodeficiency virus
IQR: Inter quartile range
NCHS: National centre for health statistics
OR: Odds ratios
OPT: Outpatient therapeutic care programs
RUFT: Ready to use therapeutic food
SAM: Severe acute malnutrition
SD: Standard deviation
TB: Tuberculosis
UTH: University teaching hospital
WHO: World health organization
WHZ: Weight for height Z-score
